# Nanostructured Carbon Nitride for Continuous-Flow Trifluoromethylation of (Hetero)arenes

**DOI:** 10.1021/acssuschemeng.3c00176

**Published:** 2023-03-22

**Authors:** Alessandra Sivo, Vincenzo Ruta, Vittoria Granata, Oleksandr Savateev, Mark A. Bajada, Gianvito Vilé

**Affiliations:** † Department of Chemistry, Materials, and Chemical Engineering “Giulio Natta”, Politecnico di Milano, Piazza Leonardo da Vinci 32, IT-20133 Milano, Italy; ‡ Department of Colloid Chemistry, Max Planck Institute of Colloids and Interfaces, Am Mühlenberg 1, DE-14476 Potsdam, Germany

**Keywords:** photocatalysis, flow chemistry, carbon nitride, metal-free catalysis, trifluoromethylations

## Abstract

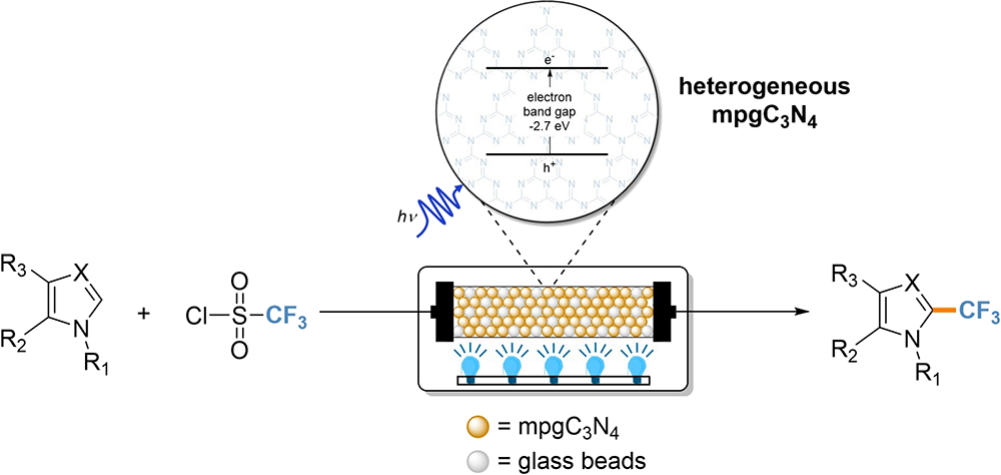

Efficient catalytic methods for the trifluoromethylation of (hetero)arenes are of particular importance in organic and pharmaceutical manufacturing. However, many existing protocols rely on toxic reagents and expensive or sterically hindered homogeneous catalysts. One promising alternative to conduct this transformation involves the use of carbon nitride, a non-toxic photocatalyst prepared from inexpensive precursors. Nonetheless, there is still little understanding regarding the interplay between physicochemical features of this photocatalyst and the corresponding effects on the reaction rate. In this work, we elucidate the role of carbon nitride nanostructuring on the catalytic performance, understanding the effect of surface area and band gap tuning via metal insertion. Our findings provide new insights into the structure–function relationships of the catalyst, which we exploit to design a continuous-flow process that maximizes catalyst–light interaction, facilitates catalyst reusability, and enables intensified reaction scale-up. This is particularly significant given that photocatalyzed batch protocols often face challenges during industrial exploitation. Finally, we extrapolate the rapid and simplified continuous-flow method to the synthesis of a variety of functionalized heteroaromatics, which have numerous applications in the pharmaceutical and fine chemical industries.

## Abstract 2

We study the interplay between the nanostructure of carbon nitride photocatalysts and the (hetero)arene trifluoromethylation performance, developing structure−performance relationships.

## Introduction

**Scheme 1:**
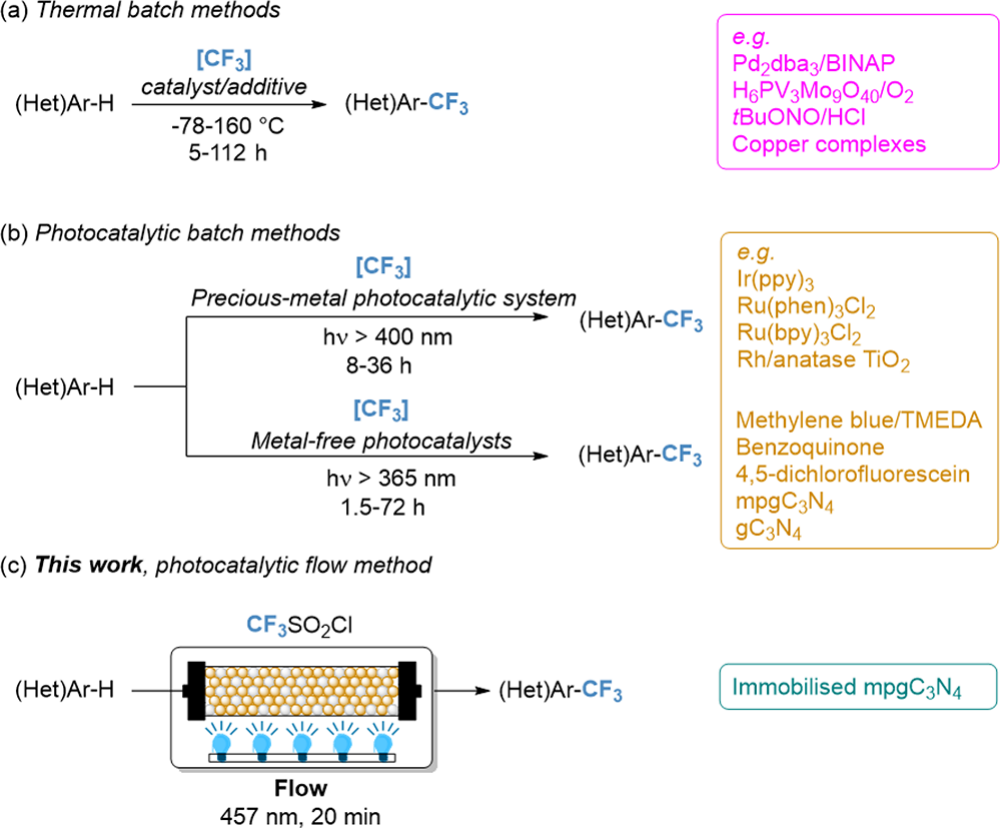
Traditional Thermal (a) and Photocatalytic (b) Batch Methods to Access Trifluoromethylated (Hetero)aromatics and the Flow Photocatalytic Strategy (c) Developed in This Work

Trifluoromethyl groups are recognized as important substituents in drug candidates, owing to their ability to enhance lipophilicity.^1−3^ Therefore, the implementation of efficient and sustainable methods for the late-stage incorporation of trifluoromethyl groups has garnered considerable attention in the field of organic and pharmaceutical synthesis.^4−6^ Conventional protocols for the thermo-catalyzed trifluoromethylation of aromatics require harsh conditions (Scheme 1a).^7−15^ In this regard, photocatalysis is an attractive technology to develop energy-saving processes that leverage light to stir chemical transformations.^16−23^ Such techniques offer numerous benefits, which include milder operational conditions (e.g., generally photochemical reactions are carried out at room temperature and atmospheric pressures),^24^ replacement of transition metals and toxic reagents with greener photoactive analogues,^25−27^ and shorter reaction times.^28^

To date, the light-driven introduction of CF_3_ to (hetero)aromatics has predominantly been achieved through metal-based homogeneous and heterogeneous photocatalysts (Scheme 1b).^29−34^ Metal-free methodologies have been suggested in the scientific literature, but their industrial implementation has been limited. This limitation has been attributed to insufficient understanding of the catalytic mechanism and difficulties associated with scaling up the methods.

Carbon nitride (C_3_N_4_) is one of the potential catalysts for metal-free trifluoromethylation.^35,36^ This non-toxic material is prepared from cheap precursors (e.g., urea, thiourea, cyanamide, dicyanamide, or melamine) via simple synthetic routes including thermal or photochemical polymerization.^37−39^ Properties of this class of materials include visible-light absorbing capabilities due to their semiconducting nature and corresponding band gap energy of ca. 2.7 eV, excellent thermo- and photostability, and tunable surface area achievable through different synthetic methods that lead to the formation of allotropes. These allotropes include graphitic carbon nitride (gC_3_N_4_), exfoliated nanosheets of graphitic carbon nitride (nC_3_N_4_), and mesoporous graphitic carbon nitride (mpgC_3_N_4_).^20,40−46^

In this work, we investigate the nanostructuring of carbon nitride for the trifluoromethylation reaction of heteroaromatics, elucidating the effect of structural properties, surface area, and band gap modulation on the reaction progression. This enabled us to derive structure–property relationships that were exploited to design a continuous-flow process for the green and upscaled production of trifluoromethylated (hetero)aromatics (Scheme 1c).^50−55^ The flow protocol was then adopted for the synthesis of a variety of functionalized heteroaromatics.

## Experimental Procedures

### Catalyst Synthesis

To prepare graphitic carbon nitride, cyanamide (10 g; Sigma Aldrich, 99%) was heated for 3 h at 550 °C (heating ramp: 10 °C min^–1^) in an alumina crucible. To prepare nanosheet carbon nitride, the as-obtained gC_3_N_4_ was exposed to further thermal exfoliation at 550 °C for 3 h (heating ramp: 2 °C min^–1^). To prepare mesoporous graphitic carbon nitride, cyanamide (3 g; Sigma Aldrich, 99%) was added to SiO_2_ Ludox HS40 with 12 nm particles (7.5 g; Sigma Aldrich, 40% aqueous dispersion) and heated under stirring at 70 °C for 16 h. The resulting white solid was heated for 8 h at 550 °C in an alumina crucible (heating ramp: 2.2 °C min^–1^). The obtained material was then added to a 4.2 M solution of NH_4_HF_2_ (12 g in 50 mL of water; Sigma-Aldrich, 95%), kept under stirring for 24 h, and then centrifuged to obtain the product after three washes with water and ethanol.

### Catalyst Characterization

The specific surface areas of the prepared materials were obtained via N_2_ physisorption experiments, degassing the samples at 150 °C for 20 h and then measuring the isotherms on a Micromeritics 3Flex porosimeter at 77 K. Data were analyzed using a QuadraWin 5.05 software package applying the Brunauer–Emmett–Teller (BET) model to the adsorption isotherms for 0.05 < *p*/*p*_0_ < 0.3. The porosity and pore distribution were calculated by using the model of quenched solid density functional theory (QSDFT) for N_2_ adsorbed on carbon (assuming cylindrical pore shape) at 77 K. A Philips model PW3040/60 X-ray diffractometer was used for powder X-ray diffraction (XRD), applying Cu Kα radiation (λ = 0.15418 nm). Elemental analysis (CHNS) was performed on a Vario Micro device by combustion.

### Trifluoromethylation Reactions

**Figure 1:**
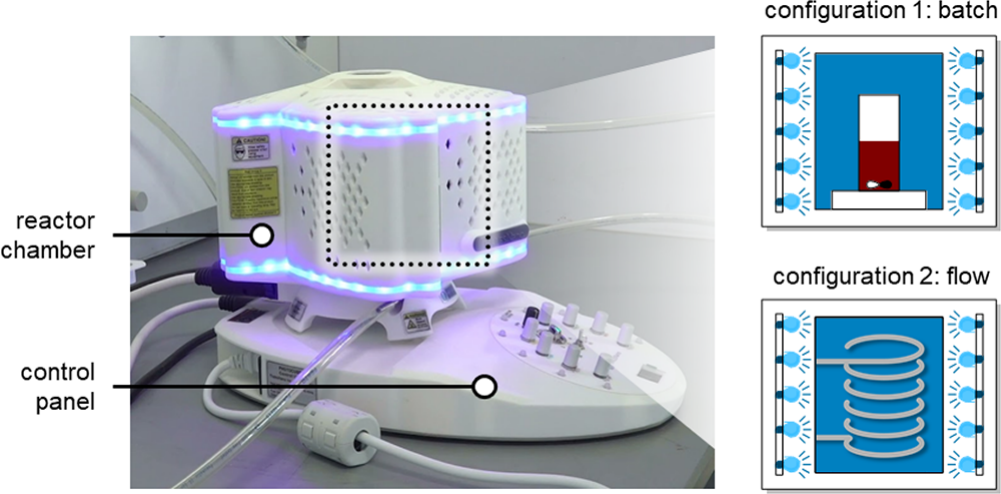
PhotoCube apparatus used for the photocatalytic experiments (courtesy of ThalesNano).

The reactions were conducted on a commercial PhotoCube apparatus, consisting of an irradiated chamber versatile enough to account both reaction flasks and flow reactors (Figure 1). The apparatus is equipped with multicolor and UV LEDs (at eight different wavelengths) with an input power of up to 128 W. Magnetic stirrers inside the chamber enable proper mixing in batch conditions.

For the batch experiments, the catalyst (100 mg) and the base (3 mmol) were added to a test tube, which was degassed for 15 min under a N_2_ flow. Then, trifluoromethanesulfonyl chloride (herein indicated as TfCl, 1.3 mmol), the (hetero)aryl compound (1 mmol), and the solvent (8 mL) were added. The reaction mixture was closed, placed in the PhotoCube apparatus, kept under magnetic stirring, and irradiated with blue light (λ = 457 nm) for 60 min. At the end of the reaction, the suspension was filtered to remove the catalyst.

For continuous-flow experiments, a physical mix of mpgC_3_N_4_ (50 mg), base (276 mg), and 50 μm of glass beads (2 g) was grinded in a mortar and blended in a vortex generator for 2 min. A transparent and flexible fluorinated ethylene-propylene (FEP) tube (500 mm long, 3.2 mm o.d., and 2.1 mm i.d.) was filled with the heterogeneous solid mixture and plugged with a quartz wool filter. The reactor was connected via 1/8″ o.d. 1/4″ 28 flat bottom flangeless fittings to the other 1/16″ o.d. tubing. The reactor volume was calculated as dead volume using the difference between the dry packed-reactor mass and the mass of the packed reactor filled with the reaction solvent. A solution of the (hetero)aryl reagent (0.25 M in acetonitrile, MeCN, flow rate: 0.03 mL min^–1^) and a solution of TfCl (0.25 M in MeCN, flow rate: 0.03 mL min^–1^) were introduced by syringe pumps (NE-1000, New Era Pump Systems, Inc.) operating at quasi-ambient pressure into the assembled packed-bed photoreactor. The reaction mixture was irradiated with blue light.

For both batch and flow experiments, an aliquot of the reaction mixture was withdrawn, and the product formation and starting material conversion were calculated using an Agilent 1200 high-performance liquid chromatography (HPLC) instrument, equipped with a UV detector G1315D working at λ = 210 nm, and a C_18_ HypersilGOLD 5 μm 175 Å column (Thermo-Fisher). Samples were analyzed using MeCN/H_2_O 60:40 as a mobile phase with a total flow rate of 0.7 mL min^–1^ at 40 °C. ^1^H and ^19^F-NMR spectra were recorded with a Bruker 400 MHz Nuclear Magnetic Resonance spectrometer.

## Results and Discussion

### Characterization of the Carbon Nitride Catalysts

Metal-free carbon nitride materials, namely, graphitic (gC_3_N_4_), nanosheet (nC_3_N_4_), and mesoporous graphitic (mpgC_3_N_4_) carbon nitrides, possessed different surface area and porosity features.^56,57^ Porosity, pore distribution, and surface area data of the prepared materials were deduced via N_2_ physisorption experiments. Brunauer–Emmett–Teller (BET) adsorption isotherms demonstrated the non-porous nature of gC_3_N_4_ and nC_3_N_4_ materials juxtaposed with that of mpgC_3_N_4_ (Figure S1a), which bore a high surface area (157 m^2^ g^–1^). The material phase purity and crystallinity were evaluated through X-ray powder diffraction (XRD) studies. XRD diffractograms showed two characteristic diffraction peaks at *2*θ = 13 and 28°, which, in accordance with the literature, correspond to the (100) and (002) planes, respectively.^58^ The former denotes the trigonal *N*-linkage of the triazine moiety, while the latter represents the stacking of aromatic rings. No other peaks were observed, confirming the absence of other crystalline impurities (Figure S1b). Elemental analysis of the prepared samples was carried out via CHNS analysis. The C/N values were found to lie in the range of 0.61–0.67 (Table S1), close to to the ideal value of 0.7 for the basic heptazine structure of C_3_N_4_. Discrepancies from the ideal value are typically ascribed to defects originating from the thermal polymerization process.^59^ Nevertheless, the low H content values confirmed a high extent of polymerization of the cyanamide precursor, with only a low percentage of non-polymerized units carrying residual protons.^59^

### Reaction Optimization in Batches and Role of the Surface Area

These materials were first evaluated under batch conditions using the PhotoCube setup as well as pyrrole **1** and trifluoromethanesulfonyl chloride (TfCl) **2** as starting materials, K_2_HPO_4_ as base, blue light, acetonitrile (MeCN) as solvent, and carrying out the reaction for 60 min. Preliminary investigation on the effect of catalyst surface area was conducted using the gC_3_N_4_ and nC_3_N_4_ samples, which afforded the desired product in yields below 10% due to the characteristic low surface area of this kind of nanostructuring. Indeed, compound **3a** was obtained with 80% yield when mpgC_3_N_4_ was used as catalyst, in accordance with the increased surface area features of mesoporous graphitic materials. The effect of the base and reactant stoichiometry are listed in Table 1. In particular, base screening experiments show that there is a direct correlation between the base strength and the selectivity for the product obtained: with a strong base such as K_2_HPO_4_ (Table 1, entry 4; p*K*_b_ = 1.3), conversion and yield of 96 and 80% were obtained, respectively, along with a high selectivity for the monotrifluomethylated product **3a** of 84%. Conversely, employing weaker bases, namely, KH_2_PO_4_ (Table 1, entry 3; p*K*_b_ = 7.2), and KF (Table 1, entry 2; p*K*_b_ = 10.83), leads to only minor yields for the desired product **3**. Moreover, the reaction process appears to be uninfluenced by the Lewis base feature of KF, whose driving force seems to be limited by the base strength. The higher yield achieved with the stronger base is probably related to the increased re-aromatization rate in presence of these if compared to weaker base condition or no base condition. A study concerning the amount of TfCl was also conducted, with the best result being given by 1.3 equivalents (Table 1, entries 4, 5, and 6). This improved selectivity for the double alkylation product **4** in the presence of an enhanced amount of the trifluoromethylating agent (Table 1, entry 6) can be rationalized on the basis of the enhanced formation and availability of CF_3_ radicals. Thus, this parameter appears to be fundamental in discriminating between the mono- and di-trifluoromethylation products, heavily influencing the selectivity of the process.

**Table 1:**
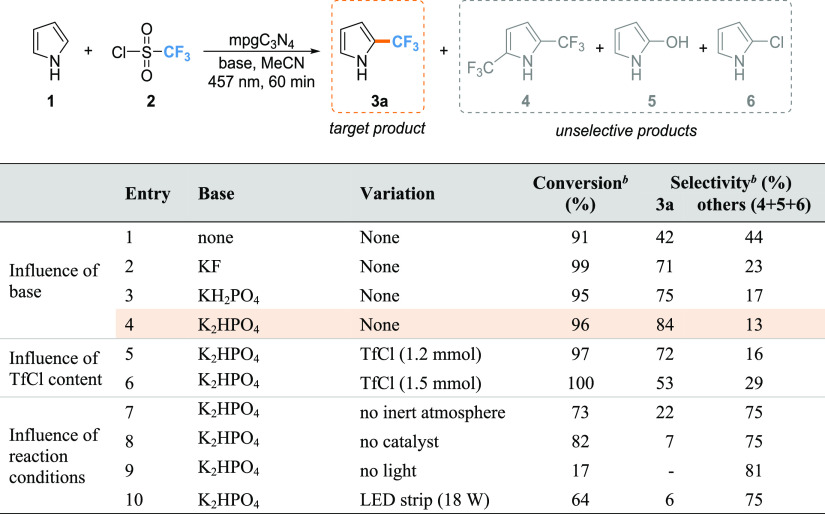
Optimization Studies for the Photocatalytic Trifluoromethylation of Pyrrole Using Mesoporous Carbon Nitride Catalyst^c^

a If not indicated otherwise, the reactions were conducted under inert conditions in a batch reactor, using **1** (1 mmol), **2** (1.3 mmol), base (3 mmol), mpgC_3_N_4_ as catalyst (100 mg), MeCN as solvent (8 mL), light wavelength λ = 457 nm (128 W), temperature = 45 °C, pressure = 1 atm, and reaction time = 60 min.

b Determined by HPLC.

c The optimal conditions are highlighted in orange.

Additional control experiments were conducted in the absence of (i) light, (ii) an inert atmosphere, and (iii) catalyst in order to determine the importance of each parameter on the model reaction (Table 1, entries 7–10). Despite the relatively high conversions obtained in the absence of an inert atmosphere and without catalyst (Table 1, entries 7 and 8), the selectivity for the desired product **3a** dropped dramatically to less than 22%. Under these two conditions, selectivity for the undesired side product **5** increased drastically (> 65%). The decreased selectivity for the trifluoromethylation product **3a** in the absence of an inert atmosphere can be accounted for through consideration of a CF_3_ radical quenching mechanism mediated by atmospheric water.^39^ Indeed, the same effect, observed when the reaction was conducted in absence of catalyst, confirms the key role of C_3_N_4_ to specifically catalyze the trifluoromethylation reaction. The importance of the irradiation conditions was confirmed by conducting the reaction in the dark (Table 1, entry 9), where it was found that a conversion of only 17% was recorded. Further studies highlighted the importance of the power of the irradiation source on the trifluoromethylation reaction: using a traditional batch setup comprised of a LED strip (18 W) wrapped around a reaction flask (see the Supporting Information for details), the conversion only reached 64%, while the selectivity for product **3a** was equal to 6% (Table 1, entry 10). The employment of the more powerful reactor (128 W) led to a beneficial outcome on the reaction performance (Table 1, entry 4), comparatively higher than the one obtained using the home-made LED setup.

**Figure 2:**
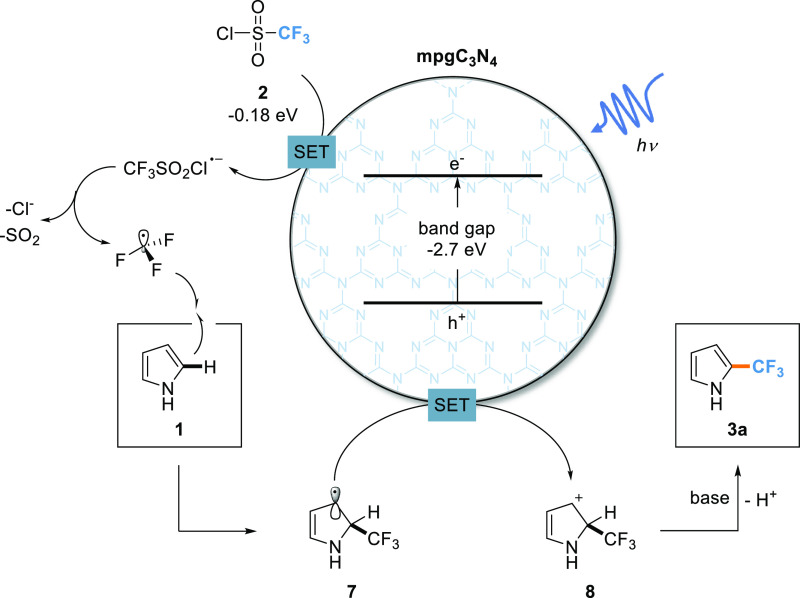
Radical mechanism for the photocatalytic trifluoromethylation of pyrrole using mesoporous carbon nitride.

A trifluoromethylation reaction mechanism has been proposed involving the generation of the radical intermediate **7** (Figure 2), in line with the literature.^32^ When the carbon nitride photocatalyst is irradiated with a photon source that exceeds the bandgap energy, the generation via single-electron transfer (SET) of a trifluoromethyl radical takes place, which selectively combines with pyrrole **1**. The catalyst-promoted oxidation of the radical intermediate **7** affords a 2-trifluoromethyl-2,3-dihydro-1*H*-pyrrolylium species **8**, which easily undergoes deprotonation in presence of the base, affording the desired trifluoromethylated arene **3a**. This elucidates the central role of the base in the reaction.

**Figure 3:**
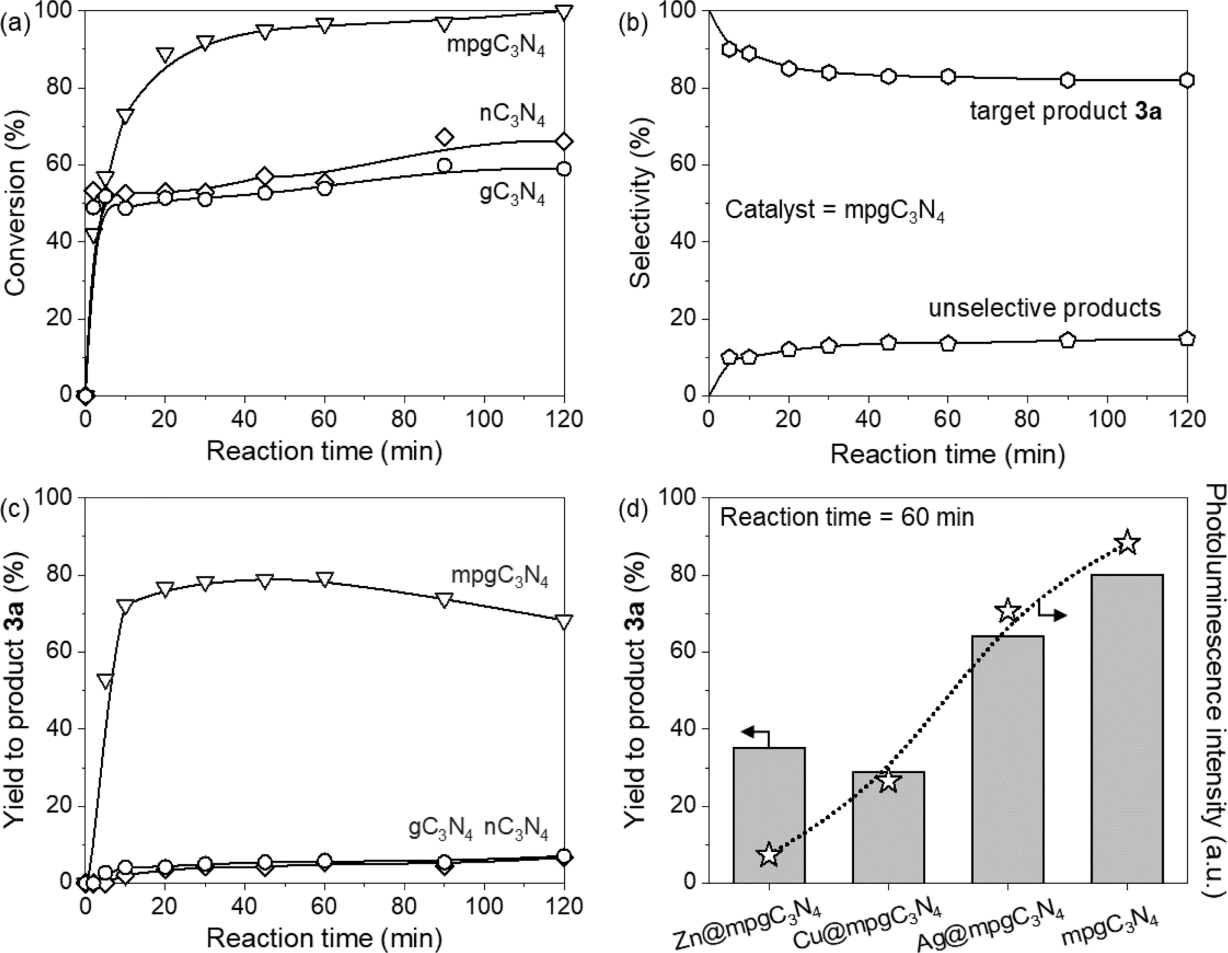
Conversion (a), product selectivity (b), and yield of target product **3a** (c, d) over various C_3_N_4_ catalysts for the trifluoromethylation of pyrrole. Reaction conditions: **1** (1 mmol), **2** (1.3 mmol), catalyst (100 mg), K_2_HPO_4_ (3 mmol), MeCN as solvent (8 mL), light wavelength λ = 457 nm (128 W), temperature = 45 °C, and pressure = 1 atm. All data are determined by HPLC. The exact distribution of the (by)products is provided in the Supporting Information.

In order to obtain a more detailed understanding of the reaction mechanism, we conducted a kinetic study on the model compound (pyrrole). The different carbon nitride analogues introduced in the previous section were thus tested, and the optimal reaction conditions in Table 1 were applied (vis-à-vis base, reactant equivalents, etc.). One of the key parameters differentiating the carbon nitrides from one another is their respective specific surface areas. Data collected from the kinetic study, conducted using the different carbon nitride catalysts with different surface areas, show a correlation between this intrinsic material property and the reaction progress (Figure 3a,b). Particularly, gC_3_N_4_ and nC_3_N_4_ appear to have a similar behavior, providing low conversion and very low yield for the desired product **3** by promoting the formation of side product **5** (cf. Table 1). A different behavior is observed for the mpgC_3_N_4_ material, which provides the desired product in high yield. An overview of the species formation trend during the kinetic study using the optimal catalyst is shown in Figure 3c. The slight decrease in selectivity for the target product is due to the slow formation of the di-alkylated side product **4**, which becomes relevant only after the first hour. The improved conversion of pyrrole and the subsequent enhanced yield for the catalytic product provided by the mpgC_3_N_4_ catalyst could be easily justified with the increase of the surface area in this material, a key parameter in the light-catalyzed heterogeneous reaction, and considering that the optical properties in the three metal-free materials, i.e., band gap and charge separation, are unaffected by the change of the tridimensional structure. Particularly, the mesopores present in the mpgC_3_N_4_ structures generates a tridimensional structure with enhanced surface area (>140 m^2^ g^–1^) if compared with the 2D graphitic (5 m^2^ g^–1^) and nanosheet (72 m^2^ g^–1^) samples; this structural effect leads to an increased contact between light, catalyst, and reactant, which is beneficial for the reaction outcome.^60^ More detailed information regarding the formation of each side product in time is provided in the Supporting Information.

### Effect of the Catalyst Band Gap on the Trifluoromethylation Reaction

Given the excellent performance of mpgC_3_N_4_, we decided to use this photocatalyst to study the effect of band gap modulation on the catalytic activity, through the introduction of isolated metal centers.^61^ In the literature, these metal-doped materials have shown enhanced catalytic properties for a number of energy-related reactions^62^ and are starting to find applications in organic synthesis.^63,64^ Metal insertion into the C_3_N_4_ framework leads to the creation of a joint electronic structure, which can in turn modify the band gap energy values, correlated also to the optical properties of the material.^65^ By fine-tuning this property using metal atoms with different electronic properties, it is possible to precisely engineer the band gap value, which, for mpgC_3_N_4_, is around 2.7 eV (CB = −1.3 eV; VB = 1.4 eV).

The presence of metal single atoms (specifically Zn, Cu, and Ag; metal-doped catalyst characterization is reported in Figures S3 and S4 and Table S2) appeared to have a detrimental effect on the yield of the desired product **3** (Figure 3d). Specifically, a similar reaction progress was observed with the Zn- and Cu-based carbon nitrides, wherein a first predominant selectivity for the side product **5** was observed followed by a slow increase for the product **3** (steeper gradient with the Cu-based material), which reached a value of 34% within 120 min. This led to overall yields of the desired product **3** that were far less superior to the undoped carbon nitride (42 and 34% for Zn- and Cu-based catalysts, respectively, versus 68% for mpgC_3_N_4_; Figure 3d). On the other hand, the Ag-doped material displays a trend in both the conversion and yield that is quite similar to that of mpgC_3_N_4_.

The following observations, when considered cumulatively, could explain the poorer performance of the Zn- and Cu- doped materials to yield the desired trifluoromethylated product **3** versus the bare mpgC_3_N_4_ support. First, the quenching of the photoluminescence (PL) spectra indicates the transfer of excited electrons to the single-atom sites (Figure 3d and Figure S3), in which the quenching follows the trend Zn@mpgC_3_N_4_ > Cu@mpgC_3_N_4_ > Ag@mpgC_3_N_4_ > mpgC_3_N_4_. Second, although the reaction vial is degassed prior to sample irradiation, a large quantity of the respective photocatalyst is employed (100 mg). It has been discussed on several counts in the literature that the trapping of O_2_ molecules within the mesoporous network of mpgC_3_N_4,_ due to its incomplete elimination via simple N_2_ degassing techniques, is indeed quite likely.^66,54^ This residual presence of O_2_ adsorbed by the mesoporous structure, coupled with the improved activation of molecular O_2_ by metal single-atoms, could yield a higher concentration of reactive oxygen species (ROS) within the local environment.^67,68^ Thus, the formation of this intermediate could in turn explain the increased selectivity for the hydroxylation of pyrrole (generating side product **5**) when Zn@mpgC_3_N_4_ and Cu@mpgC_3_N_4_ are employed in accordance with the enhanced electron transfer to the respective metal site in these materials.

### Development and Optimization of a Scalable Continuous-Flow Process

**Figure 4:**
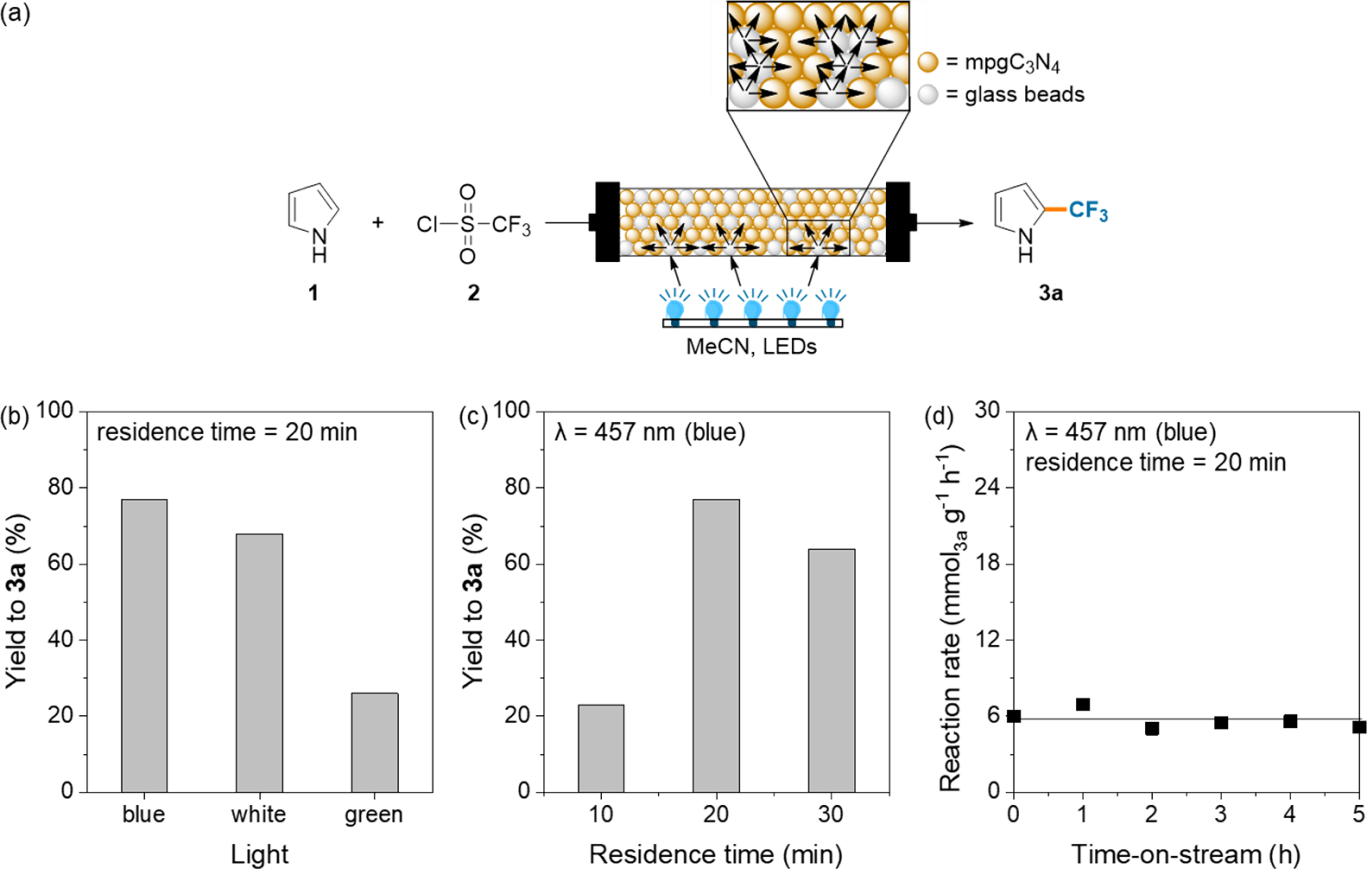
General scheme for the continuous-flow trifluoromethylation of pyrrole (a), effect of light source wavelength (b), effect of residence time (c), and catalyst stability study (d). Reaction conditions for the flow reaction: **1** (1 mmol, 0.25 M in MeCN), TfCl (1.3 mmol, 0.25 M in MeCN), various light wavelengths (blue λ = 457 nm, green λ = 550 nm, or white 400–700 nm), temperature = 45 °C, pressure = 1 atm. All data are determined by HPLC.

Succeeding the batch investigations that provide novel detailed information about the structure–performance relationship, the design of a sustainable processes was performed, developing a continuous-flow route for the trifluoromethylation process (Figure 4) in which the photocatalyst was housed within a fixed-bed reactor. The photoreactor setup was assembled using a transparent FEP tube (length = 500 mm, i.d. = 2.1 mm), which was packed with mpgC_3_N_4_, K_2_HPO_4_, and glass beads (2.5 wt %). The use of the latter as a co-packing material in the bed reactor facilitated catalyst particle separation and dilution, which was necessary to reduce the competition for the absorption of visible-light photons. Evidence supporting this positive effect can be found by conducting the reaction in absence of glass beads and using sand to dilute the catalyst in the reactor. The unfavorable contact with light provided in these conditions led us to obtain product **3a** in only 58% yield. At first, we decided to optimize the reaction protocol starting from the evaluation of the light wavelength effect on the model reaction (Figure 4a). The better performance of the catalyst in the presence of blue light (457 nm) is due to the proximity of the onset of absorption band of C_3_N_4_ with this wavelength. To investigate the reaction progress in continuous-flow mode, we evaluated the formation of trifluoromethylpyrrole **3a** screening different residence time conditions (Figure 4b). From 10 to 20 min, a reasonable yield increase was observed (respectively 23, 45, and 77%), followed by a subsequent decrease of up to 30 min (67 and 64%) related to the enhanced formation of the di*-*trifluoromethylated pyrrole **4** at longer reaction times. Choosing 20 min as the optimal residence time, comparative data between the batch and flow process with this reaction time show a similar yield between the two approaches (Table S3); the real advantages related to the continuous-flow protocol are the easier recyclability of the catalyst and the productivity enhancement. The stability of the heterogeneous photocatalyst was evaluated by running the continuous-flow reaction over 5 h on stream (Figure 4c). This has been carried out under kinetic conditions, prolonging the injection time of the solutions and evaluating the yield variation in time. The result demonstrates the excellent stability of mpgC_3_N_4_ with no activity loss, also confirmed by further characterization of the material after use (see the Supporting Information). It must be remarked that, given that the residence time of the fluid flowing through the reactor is 20 min, the steady-state operation for 5 h is equivalent to 15 catalytic cycles in a batch reactor. With the optimized conditions in hand, a continuous-flow scaled-up experiment was performed for a prolonged time (5 mmol), obtaining the target product **3a** in 62% yield and with a production rate of 0.62 mmol h^–1^, doubling the batch performance (production rate in batch = 0.32 mmol h^–1^). The enhancement in the productivity under flow conditions is justified by the high light intensity in the microreactors and irradiation condition uniformity provided by this configuration compared to the poorly designed batch conditions for conducting a heterogeneous photocatalytic reaction.^16,69^

**Scheme 2:**
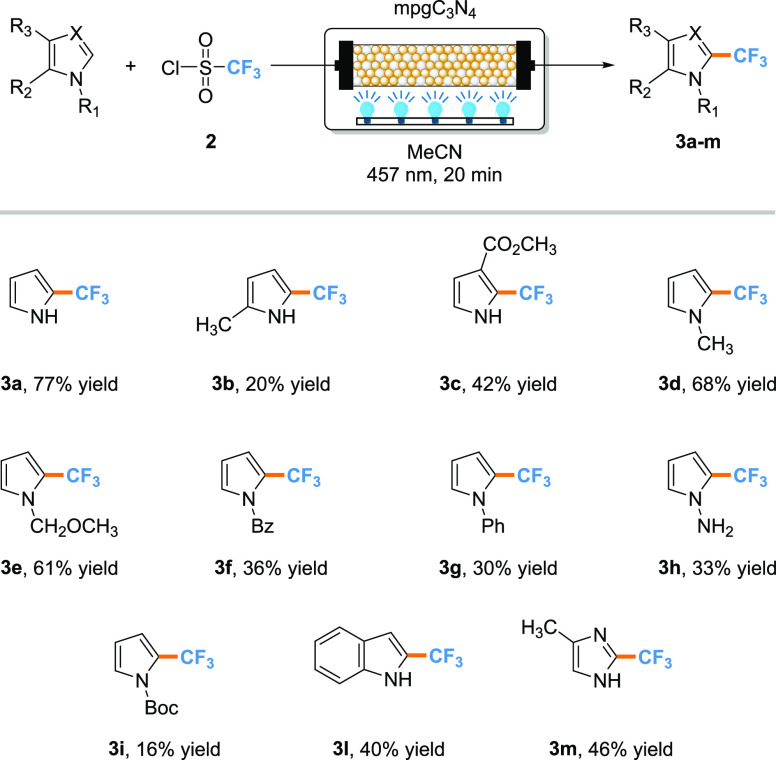
Substrate Scope for the Continuous-Flow Trifluoromethylation of (Hetero)arenes Reaction conditions: (hetero)arene (1 mmol, 0.25 M in MeCN), TfCl (1.3 mmol, 0.25 M in MeCN), temperature 45 °C, pressure 1 atm, residence time τ = 20 min. The packed-bed reactor was filled with a mix of mpgC_3_N_4_ (50 mg), K_2_HPO_4_ (3 mmol), and glass beads (2 g) to better spread the light irradiation through the catalyst bed. The yields are calculated via NMR using dibromomethane as an internal standard.

Finally, the influence of heteroaromatic reactivity and the substitution pattern toward the trifluoromethylation reaction has been evaluated using different substrates (Scheme 2). All pyrrole-based substrates performed with moderate to good yield. 2-Methyl **3b** (20% yield) and methyl-3-carboxylate **3c** (42% yield) exhibited lower reactivity, slightly offset by the electron-withdrawing effect of the ester group in the latter. The fluoroalkylation of nitrogen-substituted pyrroles occurred with good yield (compounds **3d**–**3h**, 30–68% yield) with a particular preference for substrates bearing weak electron-donating groups due to the mesomeric stabilization of the heteroaryl radical. This is further supported by compound **3i**, in which the withdrawing effect of the protecting group led to a decrease of the reaction performance (16% yield), related to the negative impact on the heteroaryl radical stability. The protocol has been also tested on different *N*-bearing heterocycles, namely, indole **3l** (40% yield) and indazole **3m** (46%). However, in the latter cases, the reaction suffered from the possibility of multiple alkylation positions with the formation of regioisomers, with 12% yield for the alkylation in C3 of the indole, and 23% yield for the C4 alkylation of the indazole.

## Conclusions

In conclusion, we have studied the effect of carbon nitride structuring on the visible light-photocatalyzed introduction of trifluoromethyl moieties. A direct correlation between the product yield and surface area has been demonstrated. The material with the highest surface area (i.e., mpgC_3_N_4_) has been further fine-tuned, introducing several isolated metals within the carbon nitride lattice to modulate the catalyst bandgap. The photoluminescence characterization of the samples has shown that electron transfer from the metal site favors the generation of reactive oxygen species, resulting in competing side-reactions involving the hydroxylation of pyrrole. These insights were exploited for the development of a continuous-flow process that remains selective and stable over long reaction times. Overall, the work demonstrates the advantages of metal-free catalysis and flow reactor technology for the synthesis of pharmaceutical intermediates in an on-demand, high-throughput fashion.

## Supporting Information Available

Catalysts and characterization (^1^H and ^19^F NMR) for new compounds (PDF)
