# Supplementary material for: Nanostructured Carbon Nitride for Continuous-Flow Trifluoromethylation of (Hetero)arenes
Source: ACS Sustain Chem Eng. 2023 Mar 22;11(13):5284–92. doi: 10.1021/acssuschemeng.3c00176 (PMC10074389; doi:10.1021/acssuschemeng.3c00176)
Supplement: Supplementary file 1 — sc3c00176_si_001.pdf [file sc3c00176_si_001.pdf]

Supporting Information

Nanostructured Carbon Nitride for Continuous-Flow Trifluoromethylation of (Hetero)Arenes

Alessandra Sivo,[†,a] Vincenzo Ruta,[†,a] Vittoria Granata,[a] Oleksandr Savateev,[b] Mark A. Bajada,[a] and Gianvito Vilé[,a]*

[a] *Department of Chemistry, Materials, and Chemical Engineering "Giulio Natta", Politecnico di Milano, Piazza Leonardo da Vinci 32, IT-20133 Milano, Italy.*

[b] *Department of Colloid Chemistry, Max Planck Institute of Colloids and Interfaces, Am Mühlenberg 1, DE-14476 Potsdam, Germany.*

[†] *These authors contributed equally to the work.*

** Corresponding author. E-mail: gianvito.vile@polimi.it.*

Number of pages: 09

Number of tables: 03

Number of figures: 07

Table of contents

1. *Characterization of metal-free C_3N_4* 3
2. *Synthesis and characterization of metal-doped C_3N_4* 3
3. *Traditional photocatalytic set-up with LED stripes* 5
4. *Reaction progress with metal-doped C_3N_4 catalysts and comparison with metal-free mpgC_3N_4* 5
5. *Kinetic studies* 6
6. *Comparison between batch and flow data* 7
7. *Characterization of metal-free mpgC_3N_4 after use* 7
8. *Compound characterization* 8

1. ***Characterization of metal-free C_3N_4***

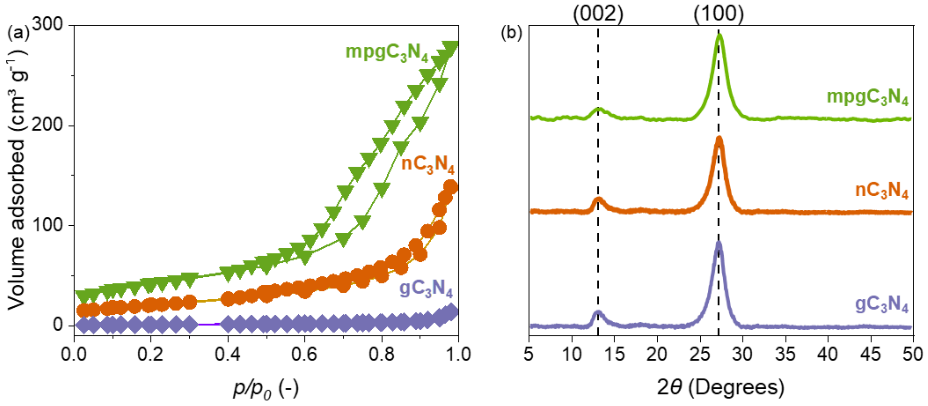

Figure S1. N_2 physisorption (a) and X-ray diffraction (b) analysis of C_3N_4-based catalysts.

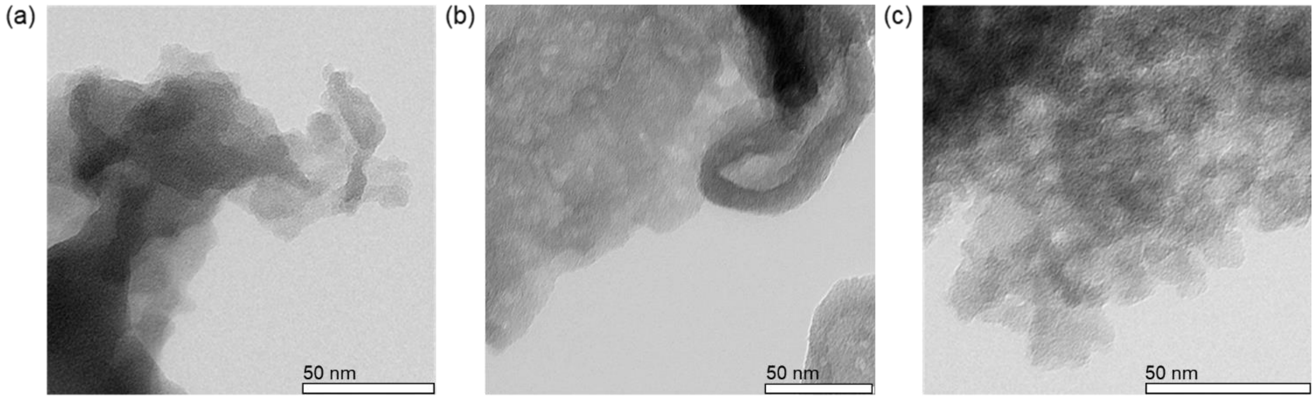

Figure S2. HRTEM analysis of gC_3N_4 (a), nC_3N_4 (b), and $mpgC_3N_4$ (c).

Table S1. Elemental composition and textural properties of C_3N_4-based catalysts.

**Catalyst**	**C[a] (wt %)**	**N[a] (wt %)**	**H[a] (wt %)**	**C/N (-)**	**S_{BET}[b] ($m^2 g^{-1}$)**	**V_{pore}[c] ($cm^3 g^{-1}$)**
gC_3N_4	34.84	56.65	1.10	0.61	5	0
nC_3N_4	32.15	47.92	2.12	0.67	72	0.21
$mpgC_3N_4$	31.90	48.75	2.43	0.65	157	0.46

[a]CHNS. [b]N_2 isotherm collected at 77 K. [c]Quenched solid density functional theory model assuming cylindrical-shaped pores.

2. ***Synthesis and characterization of metal-doped C_3N_4***

2.1 Ag, Zn, and Cu-based $mpgC_3N_4$ preparation

To prepare Zn@$mpgC_3N_4$, Cu@$mpgC_3N_4$ and Ag@$mpgC_3N_4$ materials, a solution of the corresponding chlorinated salt (0.01 mol) in 10 mL of water was stirred with a solution 1 M of sodium tricyanomethanide (1.13

g, 0.01 mol; Sigma-Aldrich, 99%) in water (10 mL) for 3 h. The resulting mixture was centrifuged to obtain the metal tricyanomethanide as white solid after three washes with water. Solid cyanamide (3 g; Sigma Aldrich, 99%) and metal tricyanomethanide (0.15 mol) were added to SiO_2 Ludox HS40 with 12 nm particles (7.5 g; Sigma Aldrich, 40% aqueous dispersion) and heated under stirring for 16 h. The resulting white solid was heated for 8 h at 550 °C into an alumina crucible (heating ramp: 2.2 °C min^{-1}). The resulting yellow solid was added to a solution of NH_4HF_2 4.2 M (12 g in 50 mL of water; Sigma-Aldrich, 95%), mixed for 24 h, and centrifuged to obtain the product after three washes with water and ethanol.

2.2 Catalysts characterization

Porosity, pore distribution, and surface area data of the metal-doped materials were deduced via N_2 physisorption experiments (**Figure S2a**). The material crystallinity was evaluated through X-ray powder diffraction (XRD) studies. XRD spectra showed two characteristic diffraction peaks at 2θ = 13° and 28°, which, in accordance with the literature, correspond to the (100) and (002) planes, respectively (**Figure S2b**). The photoluminescence (PL) spectra (**Figure S2c**) were recorded using an FP-8300 fluorescence spectrometer, setting the excitation wavelength to 365 nm.

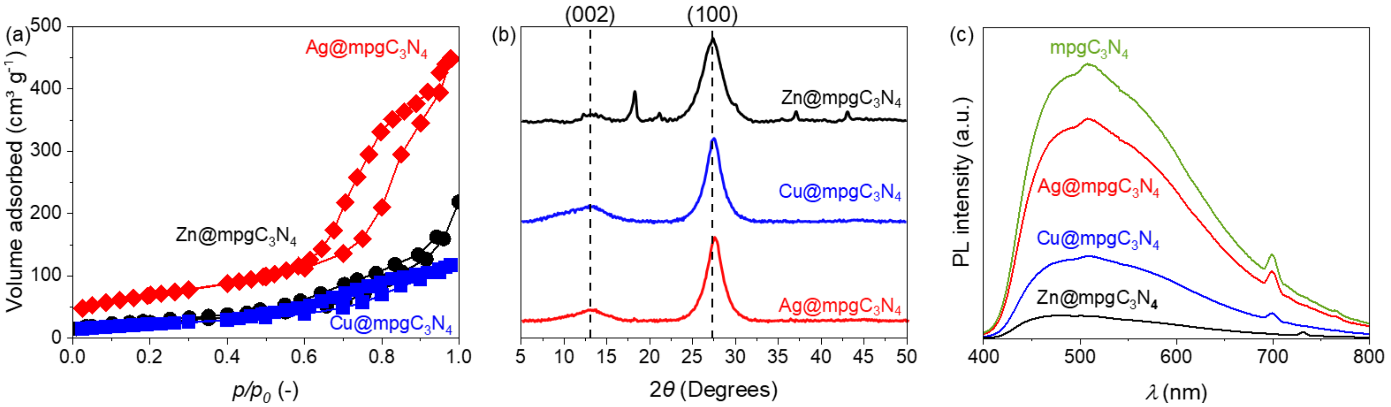

Figure S3. N_2 physisorption (a), X-ray diffraction (b), and photoluminescence (c) analysis of metal-doped C_3N_4 catalysts.

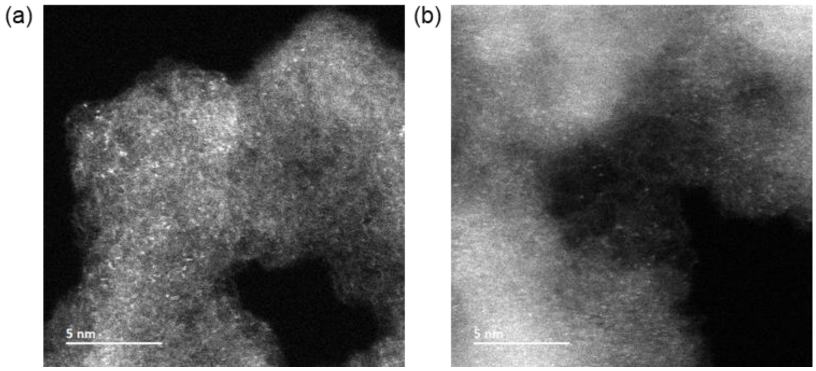

Figure S4. HRTEM analysis of Cu@mpgC_3N_4 (a) and Ag@mpgC_3N_4 (b).

The final composition and textural properties of the C_3N_4 catalysts obtained by copolymerization are shown in **Table S2**.

Table S2. Elemental composition and textural properties of C_3N_4-based catalysts.

Catalyst	C^a (wt %)	N^a (wt %)	H^a (wt %)	C/N (-)	Metal[b] (wt.%)	$S_{BET}{}^c$ ($m^2 g^{-1}$)	$V_{pore}{}^d$ ($cm^3 g^{-1}$)
Zn@mpgC_3N_4	28.38	28.38	2.75	0.54	0.65	87	0.26
Cu@mpgC_3N_4	31.37	48.78	2.20	0.64	0.50	241	0.69
Ag@mpgC_3N_4	31.01	47.43	2.40	0.65	0.30	174	0.53

[a]CHNS; [b]ICP-OES; [c]N_2 isotherm collected at 77 K. [d]Quenched solid density functional theory model assuming cylindrical-shaped pores.

3. ***Traditional photocatalytic set-up with LED stripes***

A traditional photocatalytic set-up was assembled to operate the trifluoromethylation process. A LED stripe (length = 5 m) was wrapped around a transparent bottle (i.d. = 5 cm, length = 18 cm) and the blue light (457 nm, 18 W) irradiates the reaction flask placed inside the bottle.

4. ***Reaction progress with metal-doped C_3N_4 catalysts and comparison with metal-free mpgC_3N_4***

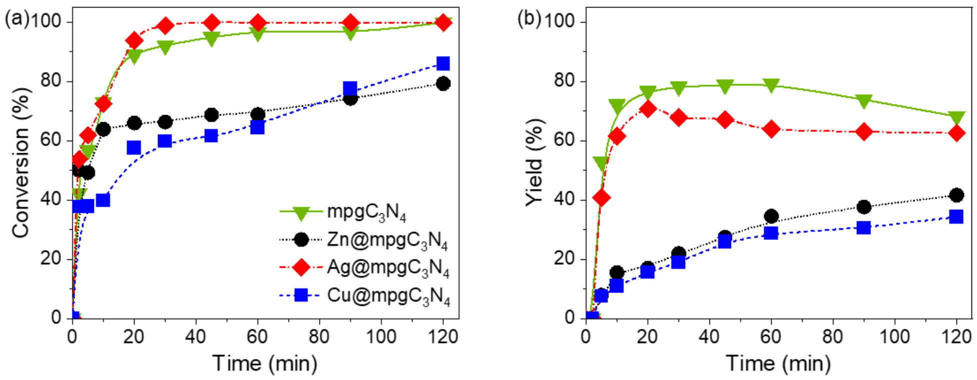

Figure S5. Synthesis of trifluoromethylpyrrole using different C_3N_4-based catalysts, showing conversion (a) and selectivity (b) trends over time. The color codes in (a) apply to (b). Reaction conditions: **1** (1 mmol), **2** (1.3 mmol), catalyst (100 mg), K_2HPO_4 (3 mmol), MeCN (8 mL), light source (PhotoCube™, blue light, λ = 457 nm), 45 °C, 1 atm.

5. Kinetic studies

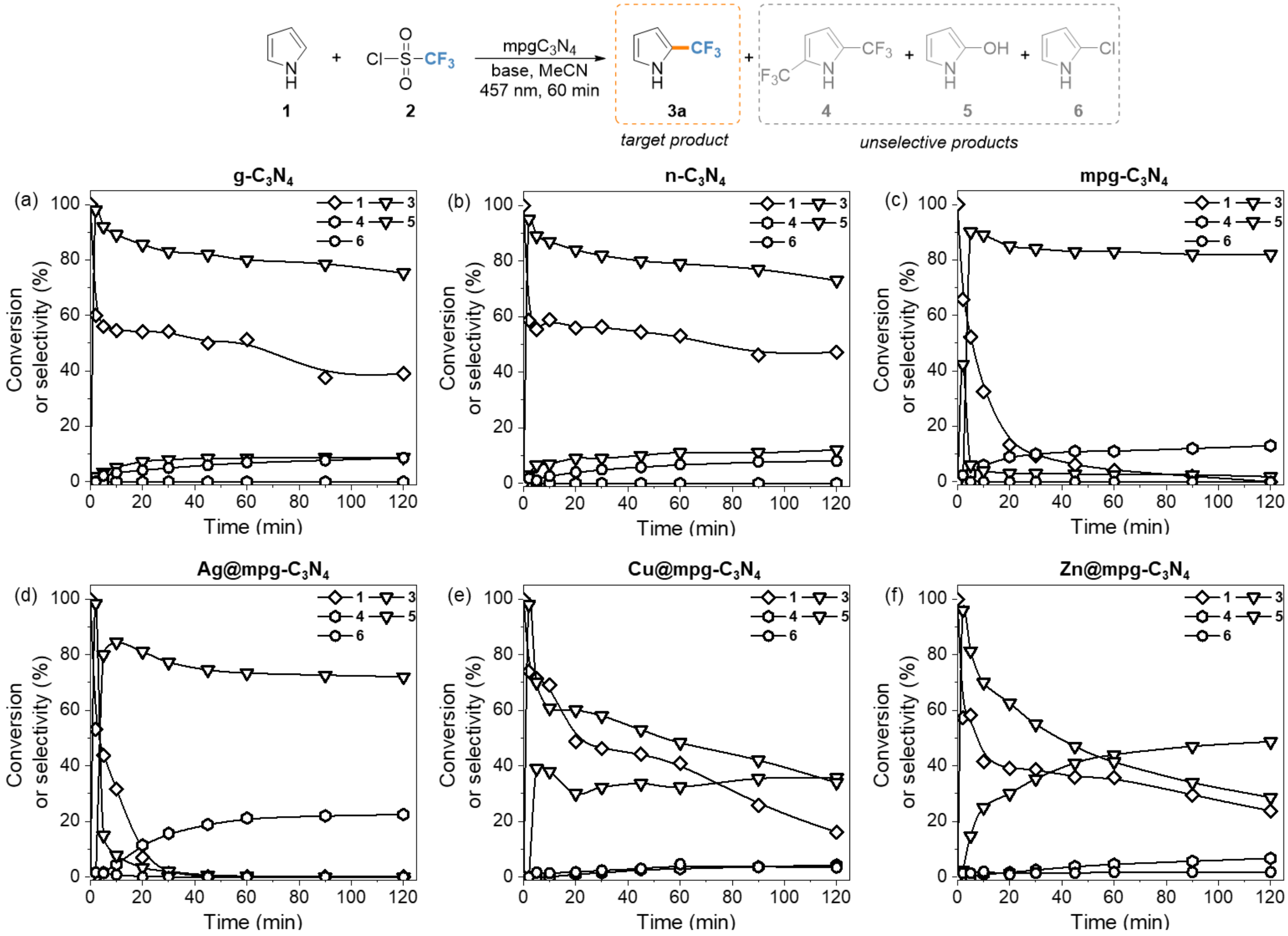

Figure S6. Synthesis of trifluoromethylpyrrole using different C_3N_4-based catalysts. Above, conversion and selectivity trends for metal-free catalysts, namely gC_3N_4 (a), nC_3N_4 (b), $mpgC_3N_4$ (c); below, conversion and selectivity trends for metal-doped catalysts, namely Ag@$mpgC_3N_4$ (d), Cu@$mpgC_3N_4$ (e), Zn@$mpgC_3N_4$ (f). Reaction conditions: **1** (1 mmol), **2** (1.3 mmol), catalyst (100 mg), K_2HPO_4 (3 mmol), MeCN (8 mL), light source (PhotoCube™, blue light, λ = 457 nm), 45 °C, 1 atm.

6. Comparison between batch and flow data

Table S3. Vis-à-vis comparison between batch and flow synthesis of trifluoromethylpyrrole.

Entry	Reaction or residence time (min)	Yield (%)	
		Batch process[a]	Flow process[b]
1	10	23	72
2	20	77	77
3	30	64	78

[a]Carried out following the general procedure for batch synthesis: **1** (1 mmol), **2** (1.3 mmol), catalyst (100 mg), K_2HPO_4 (3 mmol), MeCN (8 mL), light source (PhotoCube™, blue light, λ = 457 nm), 45 °C. [b]Carried out following the general procedure for flow synthesis: **1** (1 mmol, 0.25 M in MeCN), TfCl (1,3 mmol, 0.25 M in MeCN), light source (PhotoCube™, blue light, λ = 457 nm), 45 °C, 1 bar, packed-bed reactor filled with mpgC_3N_4 (50 mg), K_2HPO_4 (3 mmol), and glass beads (2 g).

7. Characterization of metal-free mpgC_3N_4 after use

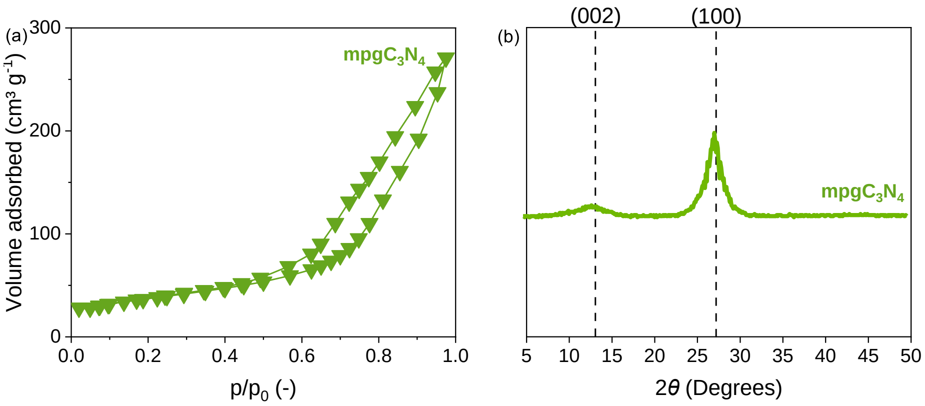

Figure S7. N_2 physisorption (a) and X-ray diffraction (b) analysis of C_3N_4-based catalysts.

8. Compound characterization

2-(trifluoromethyl)-1H-pyrrole (or “trifluoromethylpyrrole”, 3a)

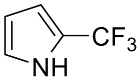

^{1}H NMR (400 MHz, CD_3CN): δ 9.80 (s, 1H), 6.97 (dd, *J* = 4.0, 2.5 Hz, 1H), 6.61 (dd, *J* = 2.2, 1.1 Hz, 1H), 6.21 (t, *J* = 10.2 Hz, 1H).

^{19}F NMR (376 MHz, CD_3CN): δ -59.51 (s).

2-methyl-5-(trifluoromethyl)-1H-pyrrole (3b)

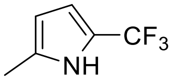

^{1}H NMR (400 MHz, CD_3CN): δ 5.85 – 5.81 (m, 1H), 5.78 – 5.73 (m, 1H), 2.19 – 2.17 (s, 3H).

^{19}F NMR (376 MHz, CD_3CN): δ -59.32 (s, *J* = 4.1 Hz).

methyl 2-(trifluoromethyl)-1H-pyrrole-3-carboxylate (3c)

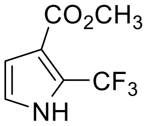

^{1}H NMR (400 MHz, CD_3CN): δ 10.92 – 10.25 (m, 1H), 6.92 (t, *J* = 2.9 Hz, 1H), 6.69 (t, *J* = 3.0 Hz, 1H), 3.81 (s, 3H).

^{19}F NMR (376 MHz, CD_3CN): δ -60.48.

1-methyl-2-(trifluoromethyl)-1H-pyrrole (3d)

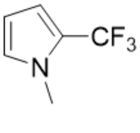

^{1}H NMR (400 MHz, CD_3CN): δ 6.88 (t, *J* = 2.0 Hz, 1H), 6.60 (dd, *J* = 2.0, 1.0 Hz, 1H), 6.14 – 6.10 (m, 1H), 3.74 (s, 3H).

^{19}F NMR (376 MHz, CD_3CN): δ -59.21 (s).

1-(methoxymethyl)-2-(trifluoromethyl)-1H-pyrrole (3e)

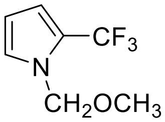

^{1}H NMR (400 MHz, CD_3CN): δ 7.12 – 7.06 (m, 1H), 6.70 (dt, *J* = 10.0, 5.1 Hz, 1H), 6.21 (dd, *J* = 7.5, 4.3 Hz, 1H), 5.31 (s, 2H), 3.26 (s, 3H).

^{19}F NMR (376 MHz, CD_3CN): δ -58.23 (s).

1-benzyl-2-(trifluoromethyl)-1H-pyrrole (3f)

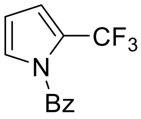

^{1}H NMR (400 MHz, CD_3CN): δ 7.42 – 7.28 (m, 3H), 7.15 – 7.09 (m, 2H), 6.97 (t, J = 2.3 Hz, 1H), 6.71 – 6.65 (m, 1H), 6.22 (t, J = 3.3 Hz, 1H), 5.26 (s, 2H).

^{19}F NMR (376 MHz, CD_3CN): δ -59.52 (s).

1-phenyl-2-(trifluoromethyl)-1H-pyrrole (3g)

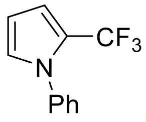

^{1}H NMR (400 MHz, CD$_3$CN): δ 7.47 – 7.42 (m, 4H), 7.41 – 7.33 (m, 1H), 7.04 (dd, *J* = 2.8, 1.9 Hz, 1H), 6.83 – 6.80 (m, 1H), 6.37 – 6.34 (m, 1H).

^{19}F NMR (376 MHz, CD$_3$CN): δ -59.52 (s).

2-(trifluoromethyl)-1H-pyrrol-1-amine (3h)

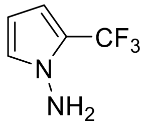

^{1}H NMR (400 MHz, CD$_3$CN): δ 6.93 – 6.86 (m, 1H), 6.49 – 6.43 (m, 1H), 6.06 – 6.00 (m, 1H).

^{19}F NMR (376 MHz, CD$_3$CN): δ -59.20.

***tert*-butyl 2-(trifluoromethyl)-1H-pyrrole-1-carboxylate (3i)**

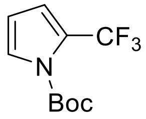

^{1}H NMR (400 MHz, CD$_3$CN): δ 7.52 (dd, J = 3.3, 1.9 Hz, 1H), 6.91 – 6.79 (m, 1H), 6.29 (t, J = 3.5 Hz, 1H), 1.62 (s, 9H).

^{19}F NMR (376 MHz, CD$_3$CN): δ -58.77.

2-(trifluoromethyl)-1H-indole (3l)

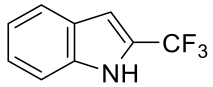

^{1}H NMR (400 MHz, CD$_3$CN): δ 7.63 – 7.58 (m, 1H), 7.54 (dd, *J* = 8.4, 0.7 Hz, 1H), 7.30 – 7.27 (m, 1H), 7.19 – 7.13 (m, 1H), 6.99 (dd, *J* = 5.8, 4.8 Hz, 1H).

^{19}F NMR (376 MHz, CD$_3$CN): δ -57.39 (s).

4-methyl-2-(trifluoromethyl)-1H-imidazole (3m)

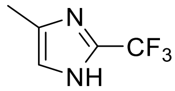

^{1}H NMR (400 MHz, CD$_3$CN): δ 8.06 (s, 1H), 7.71 (s, 1H), 7.27 (s, 1H), 2.35 (d, *J* = 1.0 Hz, 9H).

^{19}F NMR (376 MHz, CD$_3$CN): δ -60.53 (s).
